# Fast Generation of Sparse Random Kernel Graphs

**DOI:** 10.1371/journal.pone.0135177

**Published:** 2015-09-10

**Authors:** Aric Hagberg, Nathan Lemons

**Affiliations:** 1 Center for Nonlinear Studies, Theoretical Division, Los Alamos National Laboratory, Los Alamos, New Mexico, United States of America; 2 Applied Mathematics and Plasma Physics, Theoretical Division, Los Alamos National Laboratory, Los Alamos, New Mexico, United States of America; Beihang University, CHINA

## Abstract

The development of kernel-based inhomogeneous random graphs has provided models that are flexible enough to capture many observed characteristics of real networks, and that are also mathematically tractable. We specify a class of inhomogeneous random graph models, called random kernel graphs, that produces sparse graphs with tunable graph properties, and we develop an efficient generation algorithm to sample random instances from this model. As real-world networks are usually large, it is essential that the run-time of generation algorithms scales better than quadratically in the number of vertices *n*. We show that for many practical kernels our algorithm runs in time at most 𝒪(*n*(log*n*)^2^). As a practical example we show how to generate samples of power-law degree distribution graphs with tunable assortativity.

## 1 Introduction

The broad adoption of graphs as a modeling language, together with the widespread importance of applications in social, computer, and biological systems [1], has resulted in many efforts to develop random graph models [2, 3]. Random graph models give insight into network structures and are often used for null models, anonymization, and studying dynamical processes [4–7]. Large-scale graphs are also used to construct benchmarks for testing algorithm performance on high-performance computing systems [8, 9].

A common goal in constructing random graph models is to match properties of real-world graphs. One approach is to explicitly specify a distribution of graphs with expected graph properties that can be analyzed. Important examples of this approach include Erdős-Rényi random graphs [10, 11], Chung-Lu (also called expected degree) random graphs [12], and graphs with a specified degree distribution [13]. To capture even more general graph features Söderberg introduced a model of sparse inhomogeneous random graphs and showed that it could produce a wide variety of sparse graphs [14]. Bollobás, Janson, and Riordan (BJR) formalized and extended the model of Söderberg by emphasizing that the random graphs could be defined in terms of a kernel [15]. They also focused the model on sparse graphs with *O*(*n*) edges and *n* vertices. The flexibility inherent in the kernel approach generalizes well-known models of sparse graphs while remaining mathematically tractable; the BJR model can produce graphs with power-law degree distributions [15], and graphs with tunable assortativity [16].

Models from which random uniform samples can be efficiently generated are even more useful. In particular, the efficient generation of random graph instances allows researchers to simulate complex graph phenomena and dynamics for which mathematical analysis is difficult or impossible [17]. There are models, such as the space of all graphs with a given degree sequence and clustering coefficients, from which we do not know how to sample uniformly. Simulation of such models is then confined to the regions of the distribution currently available to us.

Though the BJR model appears to be so general as to preclude an efficient, general generation algorithm, we provide a fast generation algorithm for an important class of kernels, which we call Random Kernel Graphs. Random Kernel Graphs are very general and exhibit the flexibility of the inhomogeneous random graph model including tunable expected degree sequences and tunable assortativity. For Random Kernel Graphs, we exploit the idea of sampling from a waiting-time distribution to design an algorithm for generating uniform *n*-node samples with complexity of 𝒪(*n*(log*n*)^2^). We demonstrate the utility of the model by showing how to generate large sparse random graphs with a power-law degree distribution and adjustable assortativity.

## 2 Random Kernel Graphs

The BJR random graph model is extremely general, and we do not know of an algorithm which can quickly and efficiently generate such graphs. Instead, we specify an important special case of the model, the Random Kernel Graph *G*(*n*, κ) defined below, which is still very general and includes many models such as the Erdős-Rényi *G*(*n*, *p*) [11], Chung-Lu *G*(*w*) [12], and Söderberg models [14].

**Definition 1** (The Kernel κ). A non-negative, bounded, symmetric, measurable function κ: [0, 1]^2^ → ℝ is a *kernel* if there exists a finite set *D* ⊂ [0, 1] such that κ is continuous at all points (*x*, *y*) for which neither *x* nor *y* belong to *D*.

**Definition 2** (Random Kernel Graph *G*(*n*, κ)). For each positive integer *n* we define a distribution of graphs on **v**_*n*_ = {*i*/*n*:*i* = 1, 2, …, *n*}. Given a kernel κ, we define the *Random Kernel Graph G*(*n*, κ) to be the graph obtained on the vertices **v**_*n*_ when edges (*v*_*i*_, *v*_*j*_) are chosen independently with probability *p*_*ij*_ given by

$$p_{ij}≔\frac{\kappa (v_{i},v_{j})}{n}.$$

As κ is bounded and we are interested in the asymptotics of generating large graphs, we always assume that κ ≤ *n*.

For example if κ ≡ *c* is constant, then *G*(*n*, κ) is nothing more than the Erdős-Rényi random graph model in which each edge appears independently with probability *c*/*n*.

Note that *G*(*n*, κ) defines a sequence of graph distributions, one distribution for each integer *n*. This is convenient from several perspectives. Mathematically we can analyze the *n* → ∞ limit of the distributions; from a practical standpoint we can generate graphs at different scales which all come from the same model.

## 3 An efficient generation algorithm

When random graphs are defined through independent random variables, as is the case in for *G*(*n*, κ), one need only test (n2) random Bernoulli variables to choose a graph on *n* vertices uniformly from the distribution. But when modeling real-world networks, which are usually large and sparse, algorithms which take 𝒪(*n* ^2^) steps are prohibitively slow. To produce a sparse graph with *m* edges the ideal is to find algorithms that run in time 𝒪(*m*). Batagelj and Brandes found such an algorithm for producing Erdős-Rényi random graphs [18]. Instead of sampling consecutive Bernoulli random variables, the algorithm samples from a waiting time distribution (the Geometric distribution) to determine the next edge to be added. This method was extended to generate Chung-Lu random graphs in time 𝒪(*m*+*n*) [19].

As in the methods discussed above, our design of an efficient algorithm for *G*(*n*, κ) begins by drawing from waiting-time distributions instead of drawing *n* ^2^ Bernoulli variables. The random variable from our waiting distribution tells us exactly how many “non-edges” are skipped before the next edge is added to the graph. Suppose that in generating a graph *G* = *G*(*n*, κ) we have already determined that vertex *v*_*i*_ is adjacent to vertex *v*_*j*_. We would like to determine the next neighbor of *v*_*i*_ in the ordering of the indices. (By symmetry it is sufficient to determine only those neighbors of *v*_*i*_ whose index is greater than *i* so we can assume that *j* > *i*.) We first pick a random number *r* from the uniform distribution on (0,1], and then set *d* to the smallest positive integer such that,

$$\prod_{k=j+1}^{j+d}1-p_{ik}<r\;.$$ (1)

The next neighbor of *v*_*i*_ is then *v*_*j*+*d*_ so the edge (*v*_*i*_, *v*_*j*+*d*_) is added to the graph. If there is no such *d* with *j*+*d* ≤ *n* then *v*_*i*_ has no more neighbors and we continue by searching for neighbors of the next vertex *v*_*i*+1_.

The key to a fast algorithm for *G*(*n*, κ) is efficiently calculating the index *d* for each generated edge. To see how to approach this problem consider the following approximations,

$$\prod_{k=j+1}^{j+d}1-p_{ik}=\prod_{k=j+1}^{j+d}1-\frac{\kappa (v_{i},v_{k})}{n}$$ (2)

$$∼\exp(-\sum_{k=j+1}^{j+d}\frac{\kappa (v_{i},v_{j})}{n})$$ (3)

$$∼\exp(-\int_{j/n}^{(j+d)/n}\kappa \left(v_{i},y\right)\;dy).$$ (4)

The product in Eq (2) has been reduced to calculating the exponential of a definite integral. This formula can be computed efficiently, especially if an analytical form for the integral of κ can be found.

Instead of using the approximation Eq (4) to compute the waiting times we take a different approach and define a new random kernel model *G*′ based on this approximation. This new model can be generated exactly. We prove in Appendix A that the models *G* and *G*′ are asymptotically equivalent.

**Definition 3** (Random kernel graph *G*′(*n*, κ)). Let **v**_*n*_ and κ be given as in Definition 2. The random kernel graph *G*′(*n*, κ) is the graph obtained on the vertices **v**_*n*_ when the edges (*v*_*i*_, *v*_*j*_) are chosen independently with probability $p_{ij}^{\prime }$ given by

$$p_{ij}^{\prime }≔1-\exp(-\int_{v_{j-1}}^{v_{j}}\kappa \left(v_{i},y\right)\;dy).$$

We set the value of *v*_0_ = 0 to allow computation of the integral but no vertex *v*_0_ is added to the graph.

For the model *G*′(*n*, κ) we now have for relation Eq (1) the following equations,

$$\begin{array}{ll} \overset{j+d}{\underset{k=j+1}{\prod }}\left(1-p_{ik}^{\prime }\right) & =\overset{j+d}{\underset{k=j+1}{\prod }}\text{exp}\;\left(-\overset{v_{k}}{\underset{v_{k-1}}{\int }}\kappa \left(v_{i},y\right)\;dy\right)\\ & =\text{exp}\;\left(-\overset{j+d}{\underset{k=j+1}{\sum }}\overset{v_{k}}{\underset{v_{k-1}}{\int }}\kappa (v_{i},y)\;dy\right)\\ & =\text{exp}\;\left(-\overset{\left(j+d\right)/n}{\underset{j/n}{\int }}\kappa \left(v_{i},y\right)\;dy\right). \end{array}$$

Therefore, if *r* is sampled uniformly from (0,1], then we need to find the minimal *d* such that

$$\int_{j/n}^{(j+d)/n}\kappa \left(v_{i},y\right)\;dy>-\ln r\;.$$ (5)

Using inequality Eq (5) we present an efficient method for generating the model *G*′(*n*, κ) in Algorithm 1. To simplify the exposition we use the following notation,

$$F\left(v_{i},v_{j},v_{k}\right)≔\int_{v_{j}}^{v_{k}}\kappa \left(v_{i},y\right)\;dy\;.$$ (6)

**Algorithm 1** Fast Generation of *G*′(*n*, κ)

**Input:** : *n*, *F*

**Output:** : *G*′(*V*, *E*)

1: *V* ← {*v*_*i*_ = *i*/*n*:*i* = 1, 2, …, *n*}

2: *E* ← ∅

3: (*v*_*i*_, *v*_*j*_) ← (1/*n*, 1/*n*)

4: **while** *v*_*i*_ < 1 **do**

5:  Sample *r* uniformly from (0,1]

6:  *r* ← −ln*r*

7:  **If** *F*(*v*_*i*_, *v*_*j*_, *v*_*n*_) ≤ *r* **then**

8:   $\left(v_{i},v_{j})\leftarrow (v_{i}+\frac{1}{n},v_{i}+\frac{1}{n}\right)$

9:  **else**

10:   Set *d* to smallest positive integer with *F* (*v*_*i*_, *v*_*j*_, *v*_*j*_+*d*/*n*) > *r*

11:   *E* ← *E* ∪ (*v*_*i*_, *v*_*k*_)

12:   (*v*_*i*_, *v*_*j*_) ← (*v*_*i*_, *v*_*k*_)

13:  **end if**

14: **end while**

## 4 Algorithm scaling performance

If lines 6, 7, and 10 in Algorithm 1 can be calculated in 𝒪(1) time then the entire algorithm runs in time 𝒪(*n*). To see this, note that each time the *while* loop of the algorithm executes, either an edge is added to the graph or the index of *v*_*i*_ is increased by 1. Since the index of *v*_*i*_ never decreases, the *if* statement can only execute at most *n* times in total. Thus the *while* loop executes at most *m*+*n* times. For graphs with bounded κ, which are the focus of this manuscript, *m* = 𝒪(*n*) and thus the while loop executes 𝒪(*n*) times. However, line 6 requires the evaluation of a logarithm, line 7 requires the evaluation of a definite integral, and line 10 requires a root-finding algorithm. In general these operations are not 𝒪(1) running time; the speed of the integral and root-finding algorithms depend on κ. If however, *F* and its roots can be calculated in time 𝒪(1), then the entire algorithm runs in time 𝒪(*n*).

If numerical integration or root-finding is required, the complexity will be slightly worse. There are numerical integration algorithms which can calculate large classes of definite integrals in order 𝒪(log*n*) (the implied constant will depend on κ) with an error bound of *n* ^−*k*^ for fixed *k*. Root finding is also often fairly inexpensive. For large classes of functions, a root can be found in time 𝒪(log*n*) (again the implied constant will depend on κ) with an error of at most 𝒪(*n* ^−*k*^). For example if κ is a polynomial, it can be integrated analytically and Newton’s method can be used to find its roots in $O(\sqrt{\text{log}n})$ time to a precision of *n* ^−*k*^ for any fixed *k*.

**Fig 1:**
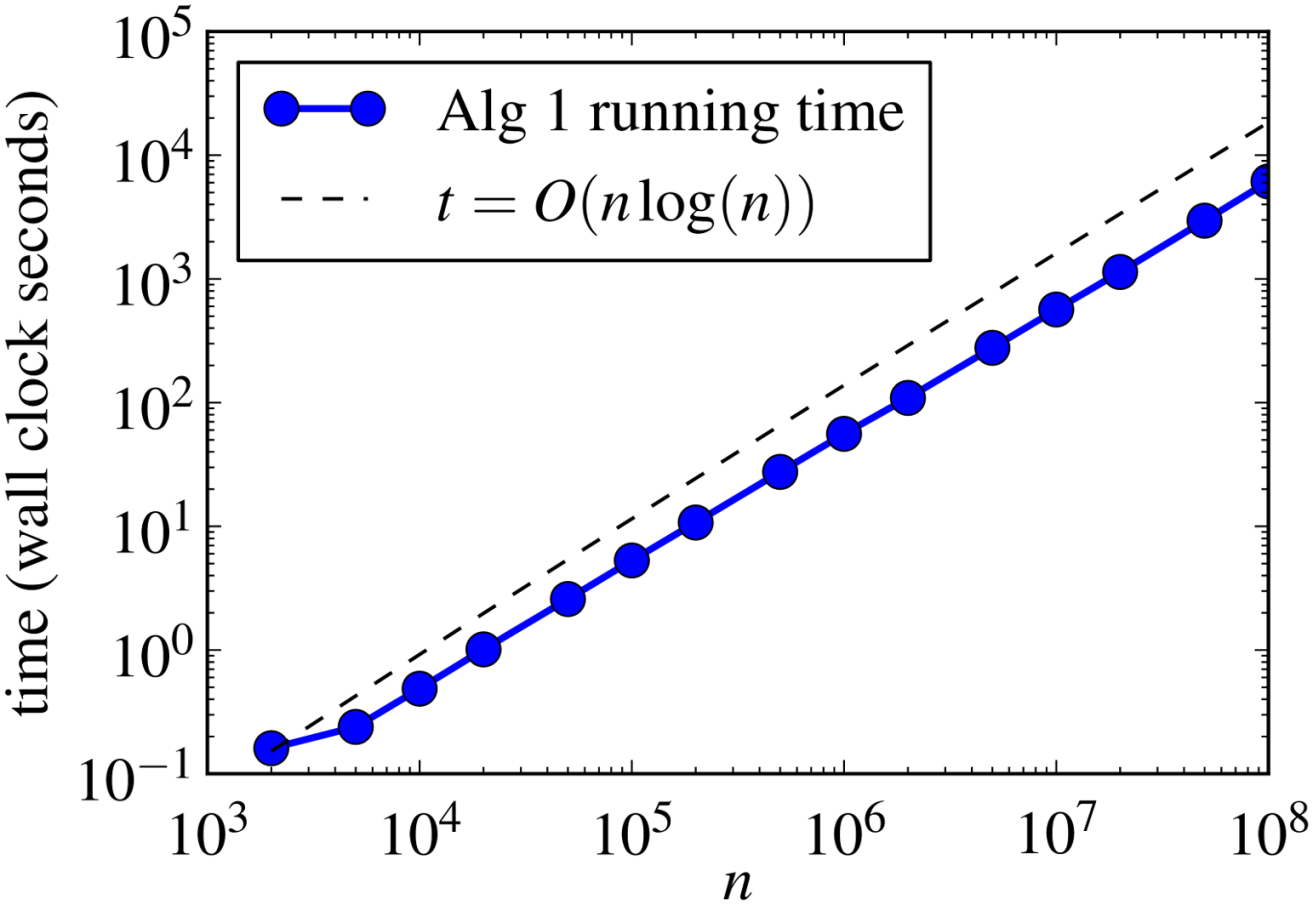
The running times for generating Erdős-Rényi *G*(*n*, *p*) random graphs κ ≡ 10 using the method in Algorithm 1. The blue circles show the average wall-clock run-time for graphs at a given *n*. The dashed reference line *t* = 10^−5^ *n*log*n* is provided to show that the average run-time performance is slightly better than the worst case estimate *O*(*n*log*n*).

We tested the scaling of Algorithm 1 by generating Erdős-Rényi random graphs with the parameter *p* = 10/*n* (κ ≡ 10) at various scales for *n* ≤ 10^8^. While it is trivial to analytically solve for the integrals and roots with constant κ, we used a numerical root solver to demonstrate that even with root solving the algorithm works efficiently. Fig 1 shows the results of our timing experiments for a Python implementation of Algorithm 1 using the NetworkX software package [20]. The data show that with numerical root finding the algorithm scales at a better (faster) rate than the worst case $O\text{(}n\sqrt{\log n}\text{).}$

Finally we note that Algorithm 1 can be trivially parallelized since the computation of the neighbors for each of the *n* vertices *v*_*i*_ can be done independently.

## 5 Generating graphs with tunable assortativity

We now provide examples for random kernel graphs with tunable assortativity, or mixing coefficients. This provides a way to generate graphs uniformly from a distribution with specified, and analytically computable, asymptotic assortativity.

### 5.1 Assortativity

The assortativity coefficient of a graph *G*, denoted ρ(*G*), is defined in the following way. Pick an edge (*u*, *v*) uniformly over all the edges in *G*. Define random variables *D*_*u*_, *D*_*v*_ to be the degrees of *u* and *v* respectively. Note that *D*_*u*_ and *D*_*v*_ by symmetry have the same distribution. Then ρ(*G*) is given by

$$\rho \left(G\right)≔\frac{\text{Cov}(D_{u},D_{v})}{\text{Var}(D_{v})}.$$ (7)

The asymptotic assortativity of *G*(*n*, κ) can be computed directly using the kernel κ [15] (see Appendix B). The formula, found in Eq (15), contains terms related to the number of copies of small subgraphs in the graph. We can use this formula to design a kernel κ with a specific asymptotic assortativity. In the following we give an example and compare the numerically computed assortativity from Eq (7) with the asymptotic value from Eq (15).

### 5.2 Power-law graphs with assortativity

To demonstrate the flexibility of the Random Kernel Graph model generator we now show how to produce graphs with a cutoff power law degree sequence and with tunable assortativity. We choose a cutoff power-law degree sequence as representative of the power-law like degree sequences which are ubiquitous in real networks [21]. For a similar reason we chose the Pearson correlation coefficient, or assortativity, as a second tunable parameter; the assortativity is widely used and its value is known for most real networks of interest [22]. The cutoff is designed to bound the maximum (expected) degree.

To generate a graph with an expected degree sequence given by a function ψ we can use the kernel κ(*x*, *y*) = *cψ*(*x*)ψ(*y*) with *c* = 1/∫_[0, 1]_ψ(*x*)*dx* (see Appendix B). To produce graphs with a cutoff power-law degree sequence we used the kernel

$$\kappa \left(x,y\right)={\left(x+.0001\right)}^{-1/2}{\left(y+.0001\right)}^{-1/2}.$$ (8)

This produces graphs such that the number of vertices of degree *k* is proportional to *k* ^−3^ up to the cutoff which occurs at *k* = 100 (*k* = 100 is the maximum expected degree). Note that in general, the kernel

$$\nu \left(x,y\right)=x^{-1/p}y^{-1/p},$$

for *p* > 0 will produce graphs with power-law like degree sequences. To see this, recall that the degree sequence will (in the asymptotic limit) follow the mixed Poisson distribution $\int_{0}^{1}\lambda (x)dx$, where $\lambda (x)=\int_{0}^{1}\nu (x,y)dy$. For large fixed *k*, we can approximate the number of vertices of degree at least *k* as follows. The measure of the set

$$\mu (\{x:\lambda (x)>k\})=k^{-p}.$$

Thus by concentration of measure, the probability a vertex will have degree at least *k* will also scale as *k* ^−*p*^. Indeed it is not hard to check that if *d*_*k*_ is the fraction of vertices with degree *k*, then

$$d_{k}∼ck^{-p-1},$$

for an appropriate constant *c* > 0 [16], Section 8.1].

The graphs produced by the kernel in Eq (8) will have expected assortativity ρ = 0; there are no degree-degree correlations. To add degree-degree correlations, we modify the kernel to

$$\kappa^{\prime }\left(x,y\right)={\left(x+.0001\right)}^{-1/2}{\left(y+.0001\right)}^{-1/2}+am_{c}\left(x,y\right),$$ (9)

where *m*_*c*_(*x*, *y*) is defined as

$$m_{c}\left(x,y\right)=\{\begin{array}{cc} 1 & \;\text{if}\;x\leq c\;\text{and}\;y\leq c\\ \frac{c^{2}}{{\left(1-c\right)}^{2}} & \;\text{if}\;x>c\;\text{and}\;y>c\\ -\frac{c}{1-c} & \;\text{otherwise.} \end{array}$$ (10)

Here, *c* is chosen in the interval (0,1] while *a* can be any nonnegative number such that κ′ is nonnegative. Note that for any such choice of *c* and *a* the expected degree sequence of κ′ is the same as that of κ since

$$\forall x,\;\forall y,\;\;\int_{0}^{1}am_{c}\left(x,y\right)dx=0.$$

Thus the term *am*_*c*_(*x*, *y*) changes the structure of the graphs *G*(*n*, κ′) without modifying their (expected) degree sequences. In particular, as κ is monotone decreasing, by modifying the parameters *a* and *c* we can produce graphs with varying degree correlations.

**Fig 2:**
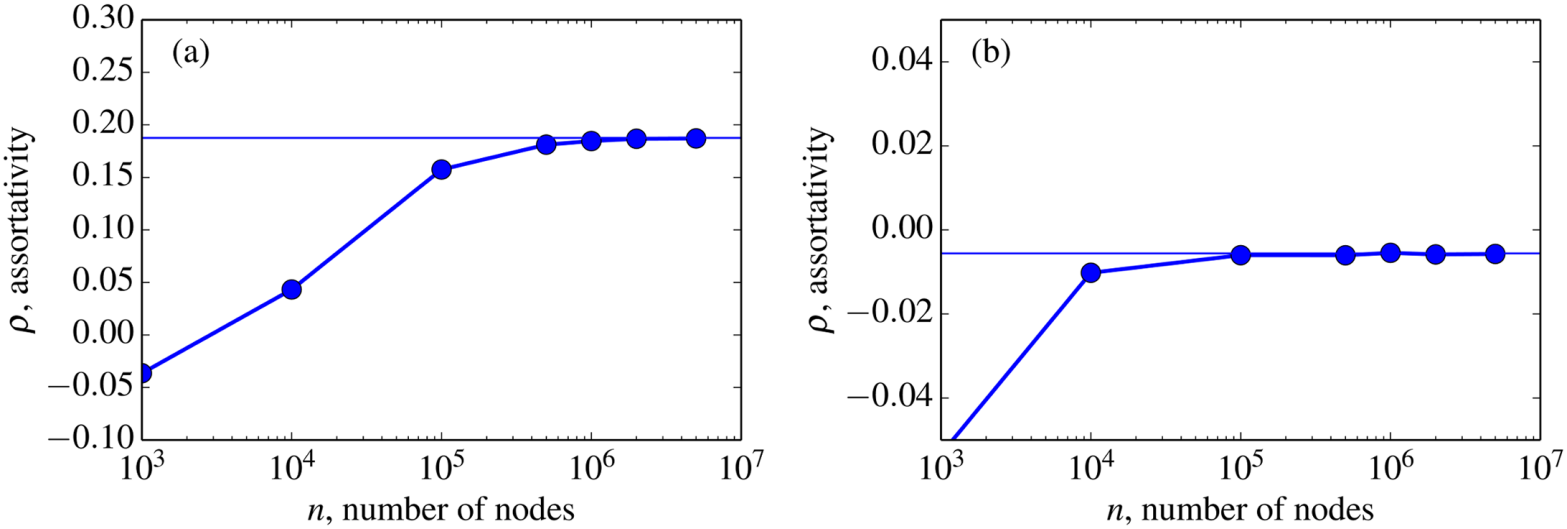
The average assortativity coefficient of graphs *G*′(*n*, κ′) generated from the kernel in Eq (8) for varying *n*. For each data point, shown by the solid circles, an ensemble of 10 graphs were generated and the average assortativity coefficient was computed for the ensemble. (a) Positive assortativity, *c* = 0.001, *a* = 30,119. (b) Negative assortativity, *c* = 0.001, *a* = −909. The horizontal line is the asymptotic calculation of the assortativity coefficient. The values converge to approximately the asymptotic value by *n* = 10^6^.

For our experiments, we chose *c* = 0.001 and then maximized or minimized *a* to produce maximal positive or negative assortativity. We used the value *a* = −909 to produce graphs with negative assortativity and *a* = 30,119 to produce graphs with positive assortativity. For these two kernels (defined by the positive and negative values of *a*), we directly calculated the expected asymptotic assortativity coefficient using the formula given in Section B. In the positive assortativity case, we calculated the asymptotic assortativity coefficient to be ρ = 0.1876, while in the negative assortativity case we calculated a value of ρ = −0.0056. The asymptotic values were then compared to averages obtained by generating multiple graphs *G*′(*n*, κ′) at varying scales and numerically calculating the associated assortativity coefficients. We have no theoretical results on the rate convergence to the asymptotic value as a function of number of vertices *n*. But the results in our experiment, shown in Fig 2, demonstrate that in this case the convergence is fast and by *n* = 10^6^ vertices has reached nearly the asymptotic value.

Despite its widespread use, the Pearson correlation coefficient is an imperfect graph statistic. Litvak and van Hofstead have shown that graphs with power-law degree sequences can only achieve non-negative assortativity in the asymptotic limit [23]. We found that even with a power-law degree sequence with a cutoff, it was hard to adjust the assortativity coefficient much below zero. Our methods however, are not necessarily exhaustive; there may indeed be graphs with cutoff power-law degree sequences that also have strongly negative assortativity coefficients.

## 6 Discussion

There have been many approaches to modeling random graphs with given properties. For example graphs with a fixed degree sequence have many applications both in pure and applied mathematics. But the search for unbiased generators of these graphs has proved quite difficult, and the general problem of efficiently generating graphs with a specified nonuniform degree sequence is still open. Even when polynomial algorithms are known to exist they can be impractical for large *n* (see [24] for a short survey). There are two main approaches to generating random graphs with a given degree sequence. The first approach, called the configuration method, was pioneered by Bender and Canfeld [25] and Bollobás [13]. Here, “stubs” on each of the vertices are matched in pairs to form the edges of the graph. This basic idea was used to define an algorithm to produce uniformly graphs with a given degree sequence in time 𝒪(*dm*) where *d*, the max degree in the graph, is restricted by *d* = 𝒪(*m* ^1/4−ε^) [24, 26]. A second approach is to use a double edge-swap operation to define a Markov chain on the space of graphs with a given degree distribution [27]. Unfortunately, it is notoriously difficult to show that these Markov chains have fast mixing. In practice various heuristics are applied to determine when to stop swapping edges [28].

Finally, we note that the model *G*(*n*, κ) will produce very few triangles. Asymptotically, the number of subgraphs *K*_3_ = 0. For some applications, such as in social networks, real-world networks have many triangles. Models that can match the triangle density or even triangles correlated with the graph degree are important [29]. It is possible to extend the *G*(*n*, κ) model and algorithm to use kernels that will produce triangles and or other subgraphs of interest. Indeed the more general BJR model has already been extended in this way [16]. Again one could specify a suitable subclass which would produce inhomogeneous random graphs with tunable clustering. The generation algorithm would then involve evaluating certain two-dimensional kernels (as well as the one dimensional case treated here). The detailed description and analysis of such and algorithm is beyond the scope of this paper and we leave it for future studies.

## A Model equivalence

Though the two models *G*(*n*, κ) and *G*′(*n*, κ) appear slightly different, we now show that they are asymptotically equivalent under realistic assumptions. Recall that the power of the model *G*(*n*, κ) comes from the fact the model can be related to the kernel κ. Specifically, many asymptotic properties can be computed directly using the kernel. The same statement holds for the variant model *G*′(*n*, κ); asymptotic properties of the graphs can be computed from κ. Given this, it is natural to expect that the two models, *G*(*n*, κ) and *G*′(*n*, κ), are in some sense equivalent in the asymptotic limit, and we now prove this.

Asymptotic equivalence is defined in the following way [30]. Let *G*(*n*, *p*_*ij*_) and *H*(*n*, *q*_*ij*_) be two random graph models defined on **v**_*n*_ with edges chosen independently: *v*_*i*_ ∼ *v*_*j*_ with probability *p*_*ij*_ (*q*_*ij*_, respectively). These two models are *asymptotically equivalent* if for every sequence (*A*_*n*_) of sets of graphs on defined on **v**_*n*_, we have

$$P\left[G_{n}\in A_{n}\right]-P\left[H_{n}\in A_{n}\right]\to 0\;,$$

for *G*_*n*_ and *H*_*n*_ sampled form *G*(*n*, *p*_*ij*_) and *H*(*n*, *q*_*ij*_) respectively.

To show that the models *G*(*n*, κ) and *G*′(*n*, κ) are, under reasonable assumptions, asymptotically equivalent, we will make use of the following theorem.

**Theorem 1** (Janson [30]) *The models G(n, p_ij_) and H(n, q_ij_) are asymptotically equivalent if*

- $q_{ij}=p_{ij}+O(p_{ij}^{2})$ *(the implied constant is uniform over *n* and choice of *i* and *j*)*
- $\sum_{i<j}p_{ij}^{3}=o(1).$

Janson further showed that if ∫_[0, 1]×[0, 1]_κ^2^ *dxdy* < ∞, then $\sum_{i<j}p_{ij}^{3}=o(1)$ holds for the model *G*(*n*, κ). We will use this result together with Theorem 1 to show that *G*(*n*, κ) and *G*′(*n*, κ) are asymptotically equivalent when κ is bounded away from zero and has bounded derivative ∂κ/∂*x*.

**Proposition 1.** *Let* κ *be be bounded away from* 0. *Suppose also that* κ *is differentiable at all points (x, y) for x, y ∉ D and that the derivative* ∂*κ*/∂*x is bounded. Then the models G(n,* κ) *and G′(n,* κ) *are asymptotically equivalent*.

*Proof*. By symmetry, the derivatives of κ are bounded, so κ is also bounded. Thus by Janson’s work, described above, we know that $\sum_{i<j}p_{ij}^{3}=O(1)$. By Theorem 1 it is thus sufficient to show that $p_{ij}^{\prime }=p_{ij}+O(p_{ij}^{2})$. Without loss of generality, let *i* < *j*. Define *f*(*x*) as

$$f\left(x\right)≔-\int_{v_{i}}^{x}\kappa \left(x,v_{j}\right)dx=\int_{x}^{v_{i}}\kappa \left(x,v_{j}\right)dx.$$

Then applying Taylor’s Theorem to approximate *f*(*v*_*i*_−1/*n*) by *f*(*v*_*i*_),

$$\int_{v_{i}-1/n}^{v_{i}}\kappa \left(x,v_{j}\right)dx=\frac{1}{n}\kappa \left(v_{i},v_{j}\right)-\frac{1}{2n^{2}}\frac{\partial \kappa }{\partial x}\left(x^{\prime },v_{j}\right)\;,$$

for some *x*′ ∈ [*v*_*i*_−1/*n*, *v*_*i*_]. Since κ bounded away from zero and ∂κ/∂*x* is bounded, there exists a constant *C*, depending only on κ, such that $|\frac{\partial \kappa }{\partial x}(x^{\prime },v_{j})|\leq C\kappa {\left(v_{i},v_{j}\right)}^{2}$ and therefore

$$\int_{v_{i}-1/n}^{v_{i}}\kappa \left(x,v_{j}\right)dx\leq \frac{1}{n}\kappa \left(v_{i},v_{j}\right)+O\;\left({\left(\frac{\kappa \left(v_{i},v_{j}\right)}{n}\right)}^{2}\right).$$

We have shown that $I_{i,j}≔\int_{v_{i}-1/n}^{v_{i}}\kappa (x,v_{j})dx$ satisfies

$$I_{i,j}=p_{ij}+O(p_{ij}^{2}).$$

Now, by definition,

$$p_{ij}^{\prime }=1-\exp(-I_{i,j}),$$

which implies that

$$I_{i,j}\geq p_{ij}^{\prime }\geq I_{i,j}-\frac{I_{i,j}^{2}}{2}.$$

Thus indeed,

$$p_{ij}^{\prime }=p_{ij}+O\left(p_{ij}^{2}\right).$$

This asymptotic equivalence condition for the two models *G*(*n*, κ) and *G*′(*n*, κ) is quite strong and relies on the assumptions that there exists an ε with κ > ε and that κ and ∂κ/∂*x* are bounded.

## B Random Kernel Graph Characteristics

Many properties of the graph *G*(*n*, κ) can be computed directly using the kernel κ. In particular, κ determines asymptotic properties of the graphs *G*(*n*, κ) as *n* → ∞ [15].

### Degree sequences

Though the model *G*(*n*, κ) cannot generate graphs with fixed degree sequences it can generate random graphs with a given expected degree as a generalization of the Chung-Lu model [12]. To see this, note that the degree *d*_*G*_(*x*) of a vertex *x* in *G* = *G*(*n*, κ) satisfies

$$\lim_{n\to \infty }E\left[d_{G}\left(x\right)\right]=\int_{\left[0,1\right]}\kappa \left(x,y\right)dy.$$ (11)

Thus if κ is a multiplicatively separable function, i.e. can be written as κ(*x*, *y*) = ψ(*x*)ψ(*y*) for some ψ:[0, 1] → ℝ^+^, then the expected degree of a vertex *x* in *G* will be proportional to ψ(*x*),

$$\lim_{n\to \infty }E\left[d_{G}\left(x\right)\right]=\psi \left(x\right)\int_{\left[0,1\right]}\psi \left(y\right)dy.$$

The full degree distribution can be determined as follows. Let *λ*(*x*) = ∫_[0, 1]_κ(*x*, *y*)*dy*. Then in the asymptotic limit, the degree distribution will converge in probability to the mixed Poisson distribution ∫_[0, 1]_Po(*λ*(*x*))*dx* [15, Theorem 3.13].

### Subgraph density

Since the expected degree of each vertex can be determined (asymptotically), it is not surprising that the edge density can also be computed. Let *e*(*G*) denote the number of edges in *G* = *G*(*n*, κ). Then

$$\lim_{n\to \infty }\frac{E[e(G)]}{n}=\frac{1}{2}\int_{{\left[0,1\right]}^{2}}\kappa \left(x,y\right)dx\;dy.$$ (12)

In fact, it is just as easy to determine asymptotically the number of other expected subgraphs *H*. Here we give the expected number of paths and cycles in the graph; other subgraphs of interest can be similarly determined (see [15] and [16]). Let *P*_*k*_(*G*) and *C*_*k*_(*G*) denote the number of paths and cycles of length *k* in the random kernel graph *G* = *G*(*n*, κ). Then

$$\lim_{n\to \infty }\frac{E[P_{k}\left(G\right)]}{n}=\frac{1}{2}\int_{{\left[0,1\right]}^{k+1}}\kappa \left(x_{0},x_{1}\right)\kappa \left(x_{1},x_{2}\right)\cdots ,\kappa \left(x_{k-1},x_{k}\right)dx_{0}\;dx_{1}\cdots dx_{k}\;,$$ (13)

and,

$$\lim_{n\to \infty }\frac{E[C_{k}\left(G\right)]}{n}=\frac{1}{2k}\int_{{\left[0,1\right]}^{k}}\kappa \left(x_{1},x_{2}\right)\cdots ,\kappa \left(x_{k-1},x_{k}\right),\kappa \left(x_{k},x_{1}\right)dx_{1}\;dx_{2}\cdots dx_{k}\;.$$ (14)

### Assortativity

A common measure of the assortativity of a graph is Pearson’s correlation coefficient, otherwise known as assortativity. The correlation coefficient can be written in terms of the number of copies of certain small subgraphs in the graph. For a fixed subgraph *H*, let *n*(*H*, *G*) be the number of isomorphic copies of *H* in *G*. Define

$$\tau \left(\kappa ,H\right)≔\lim_{n\to \infty }\frac{1}{n}E\left[n\left(H,G\right)\right].$$

In the asymptotic limit, the assortativity coefficient of *G* is given by

$$\lim_{n\to \infty }\rho \left(G\right)=\frac{\tau \left(P_{3}\right)\tau \left(P_{1}\right)-\tau {\left(P_{2}\right)}^{2}}{3\tau \left(K_{1,3}\right)\tau \left(P_{1}\right)+\tau \left(P_{1}\right)\tau \left(P_{2}\right)-\tau {\left(P_{2}\right)}^{2}}.$$ (15)

Note that τ depends on the kernel κ but we dropped this dependence from our notation to improve readability. The graph *K*_1,3_ is the complete bipartite graph with parts of size 1 and 3, that is a star with three leaves. This derivation of the asymptotic assortativity coefficient can be found in [16]. (Note that in that derivation there is a τ(*K*_3_) term which we safely ignore since it is zero for all the graphs we study.)
